# A Cyclodextrin-Based Controlled Release System in the Simulation of In Vitro Small Intestine

**DOI:** 10.3390/molecules25051212

**Published:** 2020-03-07

**Authors:** Danni Zheng, Liuxi Xia, Hangyan Ji, Zhengyu Jin, Yuxiang Bai

**Affiliations:** 1 State Key Laboratory of Food Science and Technology, Jiangnan University, Wuxi 214122, China; zdn1994danny@163.com (D.Z.); xia_liuxi610@163.com (L.X.); 170112016@stu.jiangnan.edu.cn (H.J.); fpcenter@jiangnan.edu.cn (Z.J.); 2 School of Food Science and Technology, Jiangnan University, Wuxi 214122, China; 3 Synergetic Innovation Center of Food Safety and Nutrition, Jiangnan University, Wuxi 214122, China; 4 Wuxi Biologice, Wuxi 214100, China

**Keywords:** controlled release, small intestine, cyclodextrin-based system, in vitro simulation, β-CGTase

## Abstract

A novel cyclodextrin (CD)-based controlled release system was developed in the small intestine to control the rate of drug release, on the premise of enteric-coated tablets. The system was designed based on the enzymes exogenous β-cyclodextrin glycosyltransferase (β-CGTase) and endogenous maltase-glucoamylase (MG), wherein MG is secreted in the small intestine and substituted by a congenerous amyloglucosidase (AG). The vanillin-/curcumin-β-CD complexes were prepared and detected by Fourier transform infrared (FT-IR), thermogravimetric analysis (TGA), and differential scanning calorimetry (DSC), and host CD degradation was measured based on the glucose yield. The combination of β-CGTase and AG was also functional in the CD complex system. The variations in the concentrations of added β-CGTase, with AG constantly in excess, could effectively alter the rate of host CD degradation and guest release by monitoring glucose production and color disappearance, thus, demonstrating that guest release in the CD complex system could be precisely controlled by changing the amount of β-CGTase used. Thus, the in vitro simulation of the system indicated that a novel controlled release system, based on endogenous MG, could be established in the small intestine. The CD-based controlled release system can be potentially applied in drug delivery and absorption in the small intestine.

## 1. Introduction

The controlled release is a topic in drug delivery and bioavailability that has been discussed for a long time. It is a technology that combines drugs or other substances with carrier materials and releases these entrapped substances at a certain rate to the target site for a necessary amount of time [1]. Common carrier materials are polymer and cyclodextrin (CD) and its derivatives, and the release is controlled by temperature, ultrasound, electric field [2], pressure, pH [3], or the elimination of carrier materials.

The small intestine is the major organ involved in the absorption of drugs that are administered orally. To avoid disturbances such as that by stomach acid, capsules [4] and tablets [5] were developed. It is worth noting that enteric-coated tablets play an important role in increasing absorption of the drugs in the small intestine [6]. However, disintegration of enteric-coated tablets is difficult to control [7,8]. Until now, the rate of release was controlled mainly by altering the thickness of the wall material or choosing different materials [9,10]. Enteric-coated tablets are also costly. Thus, controlled release of drugs in the small intestine is still a challenge.

CDs are excellent host molecule for drug delivery due to their merits in oxidation resistance [11], odor removal, volatility reduction [12], stability [13], and solubility, improving the functioning of encapsulated drug molecules. However, the relatively stable structure of the inclusion complex also limits the release of the guest molecule, and natural release cannot meet the actual needs. Many studies on the physical and chemical methods to control release in such a system have been conducted. Changes in temperature [14], pressure, and ultrasonic intensity [15] can control the release of molecules from the CD complex. The studies [16,17] published thus far suggested that modification of CD was very valuable in changing the rate of drug release. However, it is difficult to achieve quantitative or time-controlled release, and the requirement of guest molecules to be released at different degrees by the same carrier materials is not satisfied.

An enzymatically controlled release system was proposed, which exhibited some positive results, such as the release of prodigiosin in the presence of α-amylase and chitosanase [18] and the lipase-controlled release of Ceftiofur·Na [19]. Some studies indicated that enzymatically controlled release was useful when starch was used as the carrier material. Since the amylase–vanillin inclusion complex is hydrolyzed by salivary amylase [20], α-amylase could be used to control the drug release of tablets made of resistant starch and pre-gelatinized waxy maize starch [21]. However, in certain situations, such as drug release in different organs, the study is still limited.

In our study, we aimed to develop a CD-based controlled release system in the small intestine, on the premise of enteric-coated tablets, using the CD complex, β-cyclodextrin glycosyltransferase (β-CGTase), and maltase-glucoamylase (MG). In this in vitro simulation system, MG, which hydrolyzes linear maltodextrin with DP2-9 into glucose [22], was substituted by amyloglucosidase (AG) that has the same activity as MG. β-CD inclusion complexes were used to mimic the release of guest molecules, and hydrolyzation of β-CD hosts was done using β-CGTase and AG. CDs were hydrolyzed to glucose, the in vivo side effects, especially nephrotoxicity and the hemolytic property of β-CD, were eliminated, and the guest molecules could be released within the prescribed time. Different controlled release conditions of the same substance could be achieved by changing the amount of β-CGTase added. Thus, this system provides us with the possibility to control the release of orally administered drugs in the small intestine precisely.

## 2. Results and Discussion

### 2.1. Substrate Specificity of Single and Dual Enzyme

**Figure 1:**
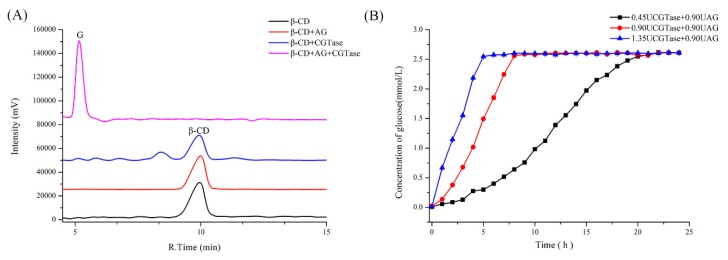
(**A**) The HPLC profile of the products obtained from β-CD by action of β-CGTase, AG, and dual enzymes and (**B**) the formation of glucose from β-CD by the action of various combinations of β-CGTase and AG.

To investigate the substrate specificity of enzymes used in the simulation system, β-CD was incubated with single β-CGTase, single AG, and dual enzymes, separately. When β-CD was incubated with single β-CGTase, only trace amounts of β-CD were hydrolyzed into smaller malto-oligosaccharides (Figure 1A) because a vast majority of the β-CD substrate was reserved due to the reaction equilibrium between the two major activities, cyclization and coupling. Meanwhile, the addition of single AG had no effect on β-CD in the same conditions. Surprisingly, on incubating β-CD with β-CGTase and AG, the resultant product was found to be only glucose, and the other oligosaccharides had disappeared. Hence, compared with the addition of single β-CGTase, the addition of AG with β-CGTase, not only hydrolyzed oligosaccharides, but also altered the dynamic balance to promoting the coupling reaction, thus, accelerating β-CD degradation.

To confirm the rate-limiting role of β-CGTase under conditions mimicking those of the small intestine with AG in excess, different amounts of β-CGTase were added. As shown in Figure 1B, when AG content remained constant, the increase in β-CGTase led to an increase in the rate of glucose release. This indicated that only when β-CGTase opened the β-CD ring, AG could use the cleaved β-CD as a substrate. It also indicated that an increase in β-CGTase accelerated the process of opening of the β-CD ring to increase the final reaction rate. Therefore, in the dual-enzyme action performance, β-CGTase was the rate-limiting enzyme in the reaction.

### 2.2. Preparation and Characterization of the Vanillin-/Curcumin-β-CD Complexes

**Figure 2:**
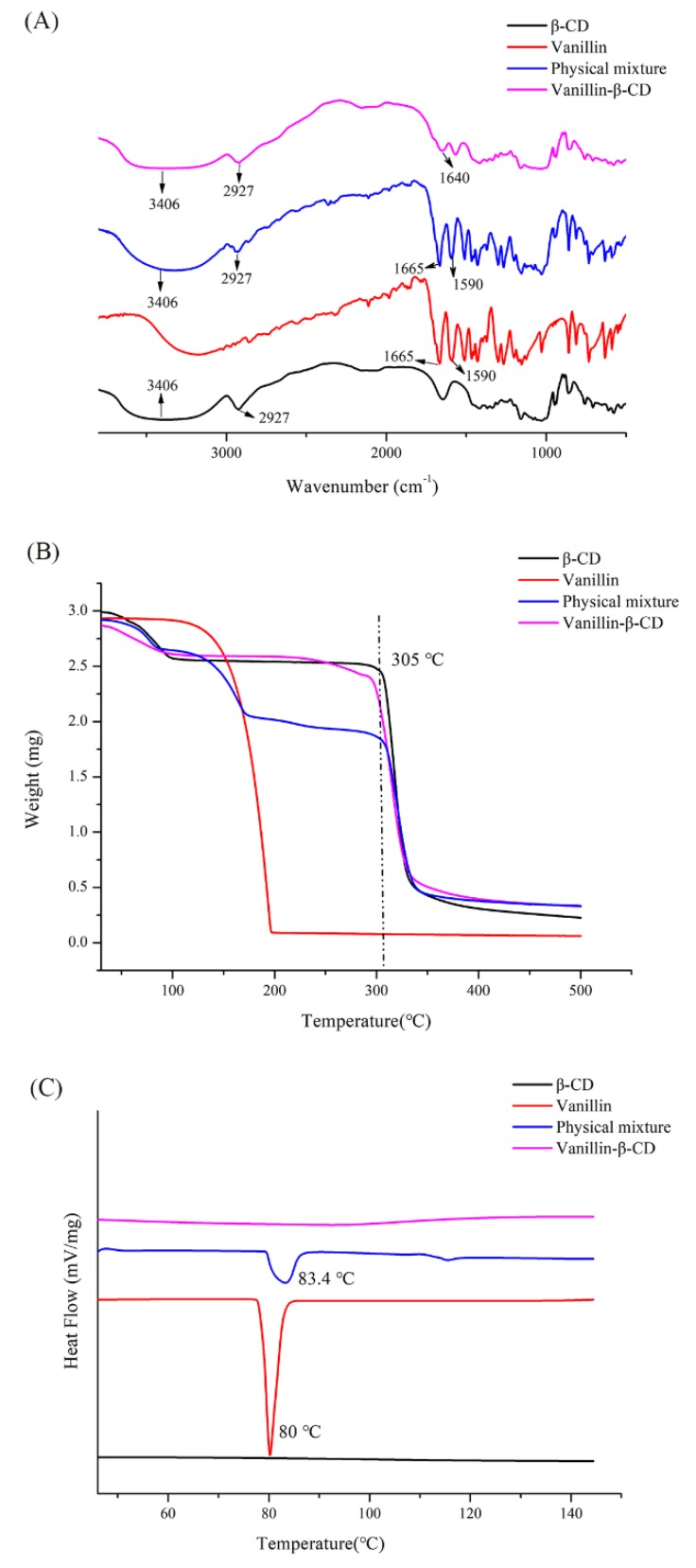
The characterization of vanillin, β-CD, the physical vanillin and β-CD mixture, and the vanillin-β-CD complex by (**A**) FT-IR, (**B**) TGA, and (**C**) DSC.

The characterization of vanillin-β-CD complex was depicted by FT-IR, TGA, and DSC. According to the results of FT-IR (Figure 2A), the characteristic peaks of pure vanillin were observed at 1590 cm^−1^(stretching absorption of benzene ring of vanillin) and 1665 cm^−1^(stretching of C=O of the aldehyde group of vanillin) [23,24]. The spectrum of β-CD exhibited characteristic peaks at 3406 cm^−1^(-OH stretching vibration) and 2927 cm^−1^(-CH2 stretching vibration). In the physical mixture, the intensity of all the characteristic peaks of vanillin was decreased. The absorption peak of vanillin at 1640 cm^−1^ was observed and reduced in intensity for the vanillin-β-CD complex. The peak of the vanillin slightly shifted for vanillin-β-CD complex because inclusion complex was probably formed by weak interactions, such as hydrophobic–hydrophobic interactions and van der Waals force, without forming new chemical bonds.

The thermal stability of vanillin, β-CD, the physical mixture, and vanillin-β-CD complex was analyzed by TGA, as shown in Figure 2B. The TGA thermograms clearly showed that vanillin completely evaporated at 200 °C, which was consistent in the literature [24]. The first zone, under 100 °C, could be related to the loss of superficial and internal water, and the second zone, around 305 °C, could be attributed to β-CD degradation. For the physical mixture, the first zone, under 100 °C, was equivalent to the evaporation of water. In addition, a slight degradation, between 100 and 200 °C, corresponded to the disappearance of vanillin. Finally, the third process, around 305 °C, was evidenced by the disintegration temperature of β-CD [25]. According to the analysis, the difference between the physical mixture and vanillin-β-CD complex could be observed. The thermal stability of the inclusion complex, compared with vanillin, was increased. The above data further proves the formation of the complex.

DSC curves of vanillin, β-CD, the physical mixture, and the vanillin-β-CD complex are shown in Figure 2C. Vanillin showed an endothermic peak at 80 °C, corresponding to its melting points [26]. This indicated that it began to change from crystalline to fluid state. The physical mixture was observed the peak of vanillin, suggesting that no inclusion process occurred. The DSC of β-CD did not show a peak in the temperature range designed. Additionally, the endothermic peak of vanillin disappeared in inclusion complexes, indicates that vanillin was protected and formed the complex successfully.

**Figure 3:**
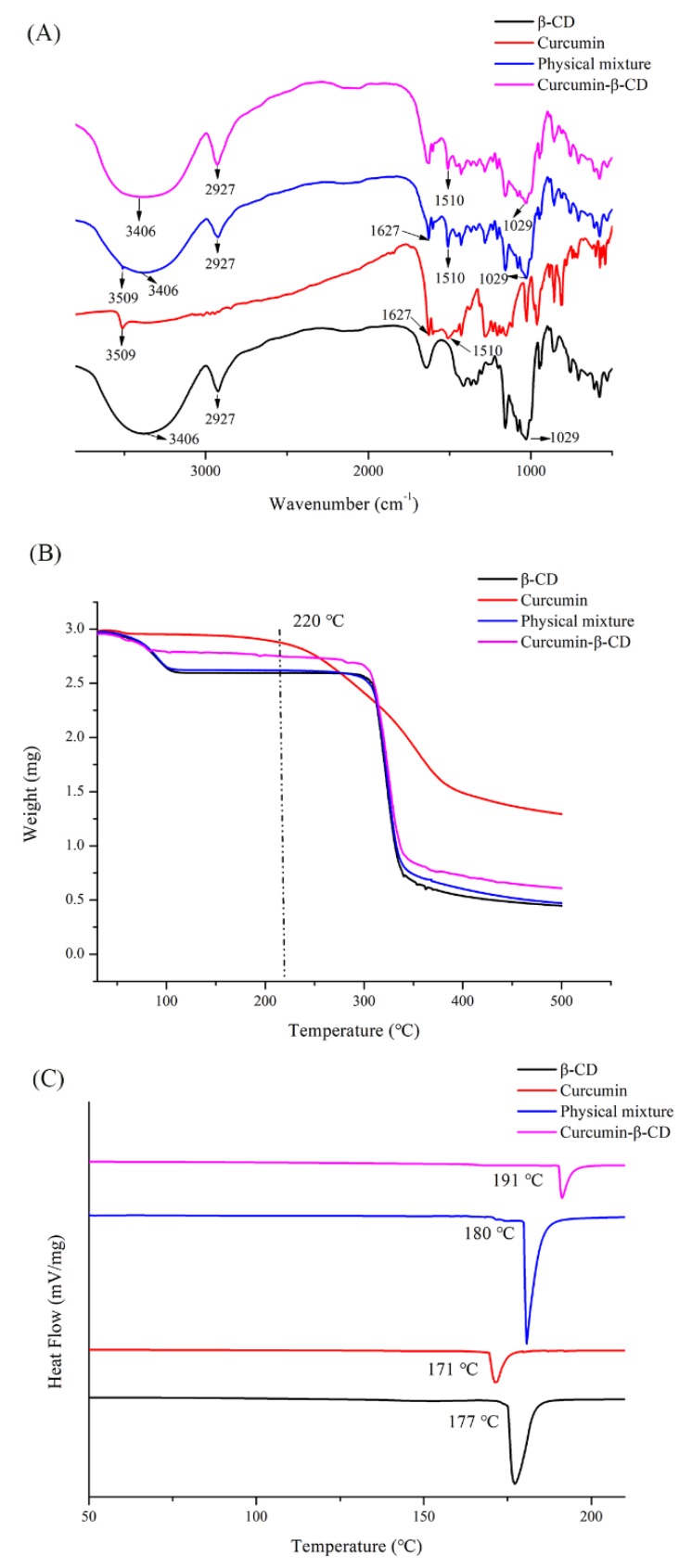
The characterization of curcumin, β-CD, physical curcumin and β-CD mixture, and curcumin-β-CD complex by (**A**) FT-IR, (**B**) TGA, and (**C**) DSC.

The same methods were used to examine the formation of curcumin-β-CD complex. The FT-IR spectra is shown in Figure 3A. The absorption band at 1627 cm^−1^ (stretching vibrations of benzene ring of curcumin) and 1510 cm^−1^ (C-O and C-C vibrations of curcumin) were exhibited. In addition, the characteristic peak of O-H stretching vibration was observed at 3509 cm^−1^, which was the same as the literature [11]. The observation of peaks in β-CD was consistent with the results of Figure 2A. However, the intensity of all the characteristic peaks was decreased in the physical mixtures, and the characteristic peaks of curcumin-β-CD almost disappeared. Therefore, the results indicated the formation of the curcumin-β-CD inclusion complex.

According to the TGA curves (Figure 3B), a slight loss of curcumin at 220 °C was also seen. The TGA thermograms of β-CD, the physical mixture, and the curcumin-β-CD complex showed that the weight loss was related to water below 100 °C. The major weight loss was around 305 °C, corresponding to the degradation of β-CD. The improvement of thermal stability of the inclusion complex was due to the interaction between curcumin and β-CD.

In addition, the thermal behavior of curcumin, β-CD, the physical mixture, and the curcumin-β-CD complex was studied using DSC, as shown in Figure 3C. The endothermic peaks of curcumin and β-CD were revealed at 171 °C [27] and 177 °C, respectively. In the physical mixture, the faint peak of curcumin and a distinct peak of β-CD were observed. For inclusion complexes, the thermogram showed an endothermic peak at 191 °C for the curcumin-β-CD complex, indicating the formation of these inclusion complexes and the resultant increase in the stability of curcumin.

### 2.3. Qualitative and Kinetic Analysis of CD-Complex Degradation by Dual-Enzyme

**Figure 4:**
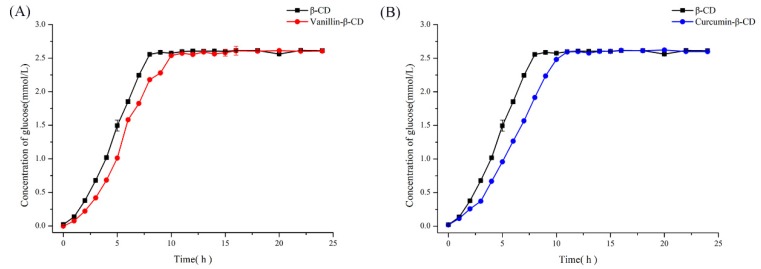
The release of glucose from (**A**) β-CD and the vanillin-β-CD complex and (**B**) β-CD and the curcumin-β-CD complex by dual enzyme action with identical amounts of β-CGTase and AG.

To estimate the dual-enzyme influence in the CD complex system, β-CD and the vanillin-/curcumin-β-CD inclusion complexes were used as substrates. β-CGTase and AG were added to an identical amount of the substrate to determine the release of vanillin/curcumin at the same time intervals by measuring the release of glucose, resulting from host β-CD degradation. Glucose release was measured because the guest vanillin and curcumin in the CD complexes showed absorbance that could influence the determination of the dissociated guest molecules. Figure 4A,B show the glucose content of the resultant product after the reaction with β-CD, vanillin-β-CD, and curcumin-β-CD. It was found that the concentration of glucose in the resultant product did not increase further after 8 h, demonstrating that all of the β-CD had been hydrolyzed to glucose. For reactions with vanillin-/curcumin-β-CD inclusion complexes, the complete release time of glucose was delayed to 10 h and 11 h, respectively. This showed that the rate of β-CD hydrolysis of the vanillin-/curcumin-β-CD inclusion complexes was slower than that of the free β-CD. This could be explained by the combination of host-guest molecules in the complexes by weak hydrogen bonds and the van der Waals force [28]. More importantly, although the rate of degradation of the inclusion complexes was slower than that of the free β-CD, the β-CD inclusion complexes could still be hydrolyzed by dual enzymes due to the equilibrium process of rapid dissociation and inclusion of the complex in an aqueous solution.

The addition of AG had no effect on the release of the guest molecules, a finding approved by our group. To confirm the amount of enzymes to be added in the controlled release reaction in the future, the kinetic data of the reaction were determined, and the results are shown in Table 1. In conditions where AG was in excess, the K_m_ and V_max_ values of β-CGTase, obtained using β-CD as the substrate, were found to be 1.39 mg/mL and 1.30 mg/(mL·min), respectively. This indicated that β-CGTase had a relatively higher affinity to β-CD than to the complexes. The K_m_ and V_max_ values of vanillin-/curcumin-β-CD complexes were 1.79 mg/mL and 0.92 mg/(mL·min) and 1.75 mg/mL and 0.88 mg/(mL·min), respectively. The k_cat_/K_m_ value of β-CD was higher than that of the vanillin-/curcumin-β-CD complexes using the same enzyme, suggesting that β-CD reacted more easily to produce glucose. This was consistent with previous results. This was found to be mainly due to the presence of guest molecules in the CD-complex system, reducing the action of β-CGTase on CD.

### 2.4. Controlled Release of Guest Molecules in the CD-Complex System

According to the results of the influence of the rate-limiting factor on CD, degradation analysis of the CD complexes, and kinetic analysis of both substrates by dual enzymes, a controlled release strategy was proposed. The rate of release was estimated by measuring both the host CD degradation and guest release.

**Figure 5:**
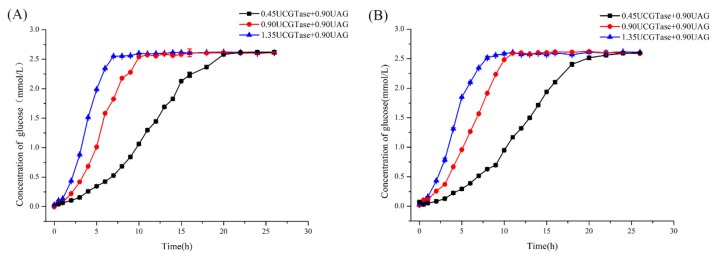
Release time of (**A**) vanillin-β-CD and (**B**) curcumin-β-CD using different amounts of β-CGTase and AG.

For this, a controlled condition was used to confirm the release time of vanillin and curcumin. The profile (Figure 5A) showed that the rate of release of glucose increased with an increase in the content of β-CGTase at the beginning of the reaction. In other words, the rate of release of vanillin constantly increased under the same conditions. When the amount of glucose (reaching 2.6 mmol/L) in the reaction no longer increased, it indicated that the host CD was fully hydrolyzed, and vanillin was released completely. Moreover, when 0.45 U, 0.90 U, and 1.35 U of β-CGTase was added to AG (0.90 U), the complete release time of vanillin was found to be 20 h, 10 h and 7 h, respectively. To verify and intuitively observe the controlled release of guest molecules from the inclusion complexes by β-CGTase and AG, the curcumin-β-CD complex was also studied. The release of glucose from curcumin-β-CD complex is shown in Figure 5B, and the result was found to be consistent with the conclusion of controlled release of vanillin. When 0.45 U, 0.90 U, or 1.35 U of β-CGTase was added to AG (0.90 U) in the reaction system, the complete release time of curcumin was 22 h, 11 h, and 8 h, respectively. Thus, the combination of β-CGTase and AG could release vanillin/curcumin from the vanillin-/curcumin-β-CD complexes, and different amounts of β-CGTase could effectively affect the release time.

**Figure 6:**
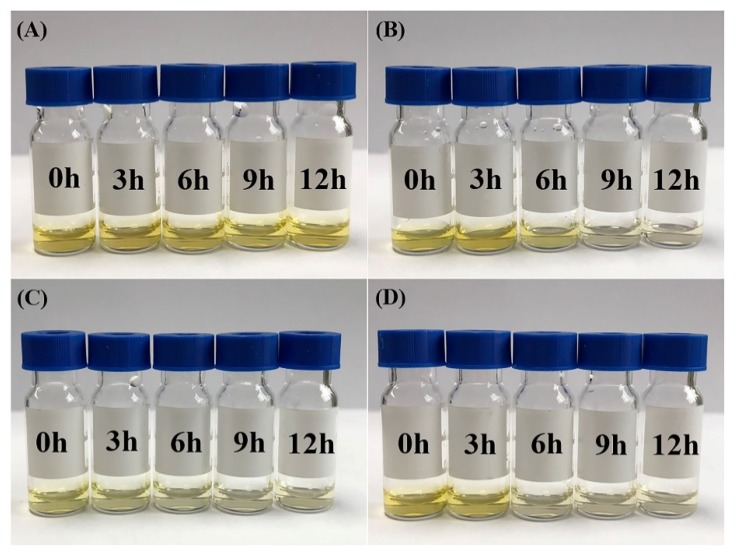
The color change of the supernatant obtained from curcumin-β-CD degradation by various concentrations of β-CGTase and AG at different times. (**A**) Curcumin-β-CD (10 mg/mL) without enzyme; (**B**) curcumin-β-CD (10 mg/mL) with β-CGTase (0.90 U) and AG (0.90 U); (**C**) curcumin-β-CD (3 mg/mL) with β-CGTase (0.90 U) and AG (0.90 U); and (**D**) curcumin-β-CD (10 mg/mL) with β-CGTase (1.35 U) and AG (0.90 U).

In addition, the guest release was estimated by visual inspection since curcumin has a certain characteristic color and is insoluble in water [29]. The released curcumin was precipitated after centrifugation, and the release of curcumin could then be directly judged by the color of the supernatant. The color change of the supernatant by the enzyme reaction at different times is depicted in Figure 6. In the reaction of the curcumin-β-CD inclusion complex without the enzyme, it was found that the color did not change substantially during 12 h of the reaction (Figure 6A). The color change of the reaction of curcumin-β-CD (10 mg/mL) with β-CGTase (0.90 U) and AG (0.90 U) is shown in Figure 6B. An obvious weakness of color was observed after 6 h, and the color of the supernatant disappeared after 9 h, suggesting that curcumin was completely released. Figure 6C reflects the effect of adding β-CGTase (0.90 U) and AG (0.90 U) to curcumin-β-CD (3 mg/mL). The color of the supernatant was significantly lighter at 3 h, indicating the faster release of curcumin. It was concluded that curcumin was completely released from the inclusion complex after 9 h because there was no color change. Figure 6C shows that curcumin was released faster from the substrate with lower concentration, when the same amount of enzyme was added. In other words, the rate of release could be adjusted by adjusting the amount of β-CGTase used. When curcumin-β-CD (10 mg/mL) was incubated with β-CGTase (1.35 U) and AG (0.90 U) for 3 h, the color of the supernatant became significantly lighter. The color then disappeared after 9 h (Figure 6D). This observation confirmed that the rate of release of curcumin was faster when more enzymes were added. This was found to be consistent with the results of Figure 6C.

In conclusion, vanillin and curcumin were used as guest molecules to investigate their controlled release by β-CGTase and AG from the CD complexes. The results indicated that the combination of β-CGTase and AG could release the guest molecules in a controlled manner, and the release time was mainly controlled by the amount of β-CGTase used.

### 2.5. Mimic Pathway of the CD-Based Controlled Release System in the Small Intestine

**Figure 7:**
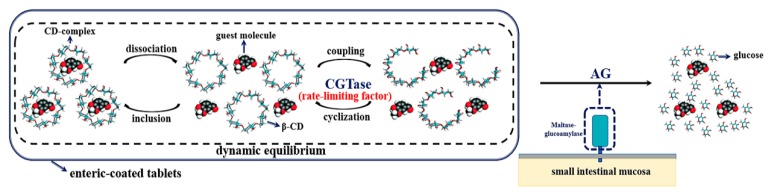
The schematic diagram of the CD-based controlled release system in the small intestine.

Based on the above results and conclusions, a mimic pathway of the CD-based controlled release system, using an enzyme that is secreted in the small intestine and another that is exogenous, was proposed. As shown in Figure 7, an enteric-coated mixture of the CD complex and β-CGTase was found to be stable, due to a dynamic equilibrium process, before reaching the small intestine. The maltase-amylase, secreted in the small intestine, altered this dynamic balance and promoted the degradation reaction, resulting in the release of guest molecules in the small intestine. The rate of release in the system was controlled by the amount of β-CGTase used. When guest molecules combined with CDs, there was no formation of ionic or covalent bonds, suggesting the presence of mainly physical effects to form inclusion complexes [30]. The CD inclusion reaction was a dynamic equilibrium process in rapid inclusion and dissociation [31]. In other words, CD, the guest molecules, and the CD complexes were present together in the system. In addition, CD was in dynamic equilibrium during β-CGTase coupling and cyclization, and the major β-CDs and minor oligosaccharides existed in this system together. In general, dissociation and inclusion, and coupling and cyclization, two pairs of processes in dynamic equilibrium, coexisted in the CD complex system of β-CGTase. MG secreted in the small intestine hydrolyzed oligosaccharides to glucose and altered the dynamic balance of the CD complex system, prompting β-CGTase to work on the formation of oligosaccharides. By using inclusion complexes, the guest molecules were effectively released and CDs were hydrolyzed to form glucose, reducing the side effects of CD. Since AG and MG have the same effects, AG was used for in vitro simulation. A series of experimental results showed that if there was no β-CGTase, the CD ring could not be opened to produce oligosaccharides, and AG could not be used for hydrolysis. In other words, β-CGTase was the key to control the release system. Hence, the release of CD-based drugs in the small intestine could be controlled mainly by the amount of β-CGTase added to achieve different release requirements.

## 3. Materials and Methods

### 3.1. Materials

β-CGTase, (EC2.4.1.19) from *Thermoanaerobacter* sp. (Toruzyme 3.0 L), was kindly provided by Novozymes Co., Ltd. (Tianjin, China). AG, (Lot 160601) from *Rhizopus* sp., was purchased from Megazyme International Ireland Ltd. (Bray, Ireland). β-CD, vanillin, and curcumin were purchased from Sinopharm Group Co. Ltd. (Wuxi, China). All other chemicals were reagent grade. GOPOD kit for glucose measurement was purchased from Beijing Leadman Biochemical Technology Co., Ltd. (Beijing, China).

### 3.2. Methods

#### 3.2.1. Enzyme Assays

The reaction mixture was composed of 1% (w/v) soluble starch (20 mM phosphate buffer solution (PBS), pH 5.5; 900 µL), having a pH similar to that in the small intestine, and β-CGTase (0.29 mg/mL; 100 µL), and the reaction was performed at 60 °C for 10 min [32]. According to the phenolphthalein method [33], the activity of β-CGTase was estimated by the decrease in absorbance at 552 nm. One unit of β-CGTase activity was defined as the amount of enzyme used for producing 1 μmol of β-CD per minute under the assay conditions.

The activity of AG was analyzed in PBS (20 mM, pH 6.0) using 1% (*w*/*v*) soluble starch as substrate at 45 °C for 10 min, according to the 3,5-dinitrosalicylic acid (DNS) method [34,35]. The reaction mixture contained the enzyme (0.17 mg/mL), and the final volume was 1 mL. DNS solution (1 mL) was then added, and the mixture was boiled for 5 min. The mixture was then cooled with ice water, and absorbance was measured at 540 nm. One unit of AG activity was defined as the amount of enzyme used for releasing 1 μmol of reducing sugars from the substrate per minute under reaction conditions.

#### 3.2.2. β-CD Hydrolysis Assays

β-CD (5.26 mg) was weighed and diluted to 10 mL using PBS (20 mM, pH 5.5). Then, β-CGTase solution (100 μL, 0.45 U) and AG solution (100 μL, 0.90 U), β-CGTase solution (200 μL, 0.90 U) and AG solution (100 μL, 0.90 U), and β-CGTase solution (300 μL, 1.35 U) and AG solution (100 μL, 0.90 U) were added separately to the inclusion complex solution (2.4 mL), which was then diluted to 3 mL. The mixture was incubated at 37 °C. At every 1 h interval, samples (100 µL) were taken, and the reaction was stopped by adding NaOH (0.4 M, 50 µL). The samples were then neutralized by adding HCl (0.4 M, 50 µL). The amount of glucose in the reaction product was determined by the GOPOD kit.

#### 3.2.3. Vanillin/Curcumin Release Assays

The complete release time of vanillin/curcumin from the β-CD complex was determined. Vanillin-β-CD (5.97 mg) and curcumin-β-CD (5.69 mg) were dissolved separately in PBS (20 mM, pH 5.5; 10 mL). The inclusion complex solution (2.4 mL) was obtained. β-CGTase solution (100 μL, 0.45 U) and AG solution (100 μL, 0.90 U), β-CGTase solution (200 μL, 0.90 U) and AG solution (100 μL, 0.90 U), and β-CGTase solution (300 μL, 1.35 U) and AG solution (100 μL, 0.90 U) were added separately to the sample, and the obtained solution was finally diluted to 3 mL. The reaction and test conditions were the same as those for β-CD hydrolysis assays. β-CD (5.26 mg) was used as reference.

Color change of the supernatant, due to the enzyme reaction, at different times was determined. The substrate, curcumin-β-CD in PBS (20 mM, pH 5.5; 10 mg/mL), and the sample (2 mL) were mixed. β-CGTase solution (200 μL, 0.90 U) and AG solution (100 μL, 0.90 U), and β-CGTase solution (300 μL, 1.35 U) and AG solution (100 μL, 0.90 U) were added separately to the above mentioned sample, and the obtained solution was diluted to a final volume of 2.5 mL. The substrate, curcumin-β-CD in PBS (20 mM, pH 5.5; 3 mg/mL) and the sample (2 mL) were mixed. β-CGTase solution (200 μL, 0.90 U) and AG solution (100 μL, 0.90 U) were added, and the obtained solution was diluted to 2.5 mL. Curcumin-β-CD in PBS (20 mM, pH 5.5; 10 mg/mL), without the enzyme, was used as reference. The reaction conditions were the same as those for β-CD hydrolysis assays. Samples were taken every 3 h. The reaction sample was centrifuged at 10,000 rpm for 5 min, and the supernatant was taken for color comparison.

#### 3.2.4. Preparation of Inclusion Complexes

The vanillin-/curcumin-β-CD inclusion complexes were prepared by the saturated solution method [36]. Briefly, vanillin-/curcumin-ethanol solutions were added to β-CD aqueous medium (10 mM) in 1:1 and 1:4 molar ratios, respectively. The resultant mixtures were stirred at room temperature for 12 h. The vanillin-/curcumin-β-CD solutions were then frozen at −80 °C and lyophilized in a labconco freeze drier (Free Zone 4.5 L, Labconco, Kansas, MO, USA) to obtain solid β-CD complexes. The powdered complexes were placed on filter paper and rinsed with ethanol to remove non-complexed vanillin and curcumin. Finally, the complexes were freeze-dried.

#### 3.2.5. Fourier Transform Infrared (FT-IR) Spectroscopy

FT-IR spectra were recorded for the study of molecular samples at wavelengths of 400–4000 cm^−1^ using an IS10 FT-IR spectrometer (Thermo Nicolet, Waltham, MA, USA). Vanillin, curcumin, β-CD, and corresponding inclusion complexes were powdered with dry KBr (1:20) to obtain transparent tablets for analyses.

#### 3.2.6. Differential Scanning Calorimetry (DSC)

The thermal behavior of vanillin, curcumin, β-CD, the physical mixtures, and the inclusion complexes was determined by DSC (SII Nano Technology Inc., Chiba, Japan). Samples (2–3 mg) were weighed and crimped hermetically with an aluminum cover. Measurements were performed at a rate of 10 °C/min from 20 to 250 °C, with the nitrogen flow at 20 mL/min and an empty aluminum capsule as the blank control.

#### 3.2.7. Thermogravimetric Analysis (TGA)

The sample (3 mg) was dispersed uniformly in the alumina crucible. TGA (instrument from Mettler Toledo Instruments Co. Ltd., Zurich, Switzerland) was performed from 30 to 500 °C to study the thermal behavior of the sample at a heating rate of 20 °C/min, with the nitrogen flow at 50 mL/min.

#### 3.2.8. High Performance Liquid Chromatography (HPLC)

β-CD (5.26 mg) was weighed and diluted to 10 mL with PBS (20 mM, pH 5.5). β-CGTase solution (100 μL, 0.45 U) and AG solution (100 μL, 0.90 U), and β-CGTase solution (100 μL, 0.45 U) and AG solution (100 μL, 0.90 U) were added separately to the obtained solution (2.40 mL), which was then diluted to 3 mL. The reaction conditions were the same as those for β-CD hydrolysis assays, and β-CD solution without the enzyme was used as reference.

The reaction samples were then analyzed by a high-performance liquid chromatograph (LC-20AT pump, RID-10A detector; Shimadzu, Japan) equipped with APS-2 HYPERSIL column (250 mm × 4.6 mm column, Thermo scientific), as previously reported [37]. After the sample was passed through a syringe filter (0.45 µm), analyses were performed at 30 °C with a mobile phase of acetonitrile/water (75:25, *v*/*v*) at a flow rate of 1 mL/min.

#### 3.2.9. Kinetics of the Release of β-CD Inclusion Complex

Varying concentrations (0.10, 0.20, 0.40, 0.60, 0.80, 1.00, 2.00, 3.00, and 4.00 mg/mL) of β-CD, vanillin-β-CD, and curcumin-β-CD were prepared separately. β-CGTase solution (100 μL, 0.45 U) and AG solution (100 μL, 0.90 U) were then added to the obtained solution. The reaction and test conditions were the same as those for β-CD hydrolysis assays, and samples were taken every 30 min.

## 4. Conclusions

A novel CD-based controlled release simulation system was developed based on enzymes, exogenous β-CGTase and endogenous MG, secreted in the small intestine. The rate of drug release from the CD-based system was controlled by regulating the amount of the enzyme added to the system. The experimental results suggested that the release of the guest molecule from the inclusion complexes within the prescribed time could be controlled by altering the amount of the rate-limiting β-CGTase used. This novel controlled release system, based on enzymes secreted in the small intestine, had the potential to control release accurately, overcoming the limitations of traditional enteric-coated tablets. Moreover, the novel CD-based controlled release system offered a means to achieve different influences on the release of the same guest molecule, and it provides a great prospect for controlling the release of volatile flavor substances and effective functional ingredients.

## Figures and Tables

**Table 1:** The kinetic parameters of dual-enzyme action on β-CD and the CD complexes.

	K_m_ (mg/mL)	V_max_ (mg/(mL × min))	k_cat_ (min^−1^)	k_cat_/K_m_ (mL × min^−1^ × mg^−1^)
β-CD	1.39	1.30	4.48	3.22
Vanillin-β-CD inclusion complex	1.79	0.92	3.17	1.77
Curcumin-β-CD inclusion complex	1.75	0.88	3.04	1.74
